# 

**Published:** 1891-09-05

**Authors:** 


					fnHDUMlEd
r for infants
IdlHllLAdd
AND INVALIDS.
No. 258. Vol. X.] Saturday,
September 5th, 1891.
[One Penny.'

				

